# Supplementary material for: Exploring factors contributing to antibiotic resistance: A cross-sectional empirical study in Bangladesh
Source: PLoS One. 2026 May 18;21(5):e0344449. doi: 10.1371/journal.pone.0344449 (PMC13183214; doi:10.1371/journal.pone.0344449)
Supplement: Attachment — Submitted filename: Response to Reviewers.docx [file pone.0344449.s001.docx]

**Response to Reviewers**

Reviewer #1: To add line number for the ease of providing comments from reviewers.

**Response:** We thank the reviewer for this suggestion. As the submission guidelines for PLOS ONE do not require the inclusion of line numbers, we have retained the current formatting in order to align with the journal’s specifications. However, we have ensured that all revisions are clearly highlighted and that section, paragraph, and table numbers are consistent throughout the manuscript to facilitate ease of reference for reviewers.

Please check citation style according to the guidelines.

**Response:** All references have been carefully reviewed and reformatted to strictly follow the citation style guidelines of PLOS ONE.

Why was the Literature Review added in detail in the manuscript? Instead of this, some salient and relevant information should be described in the introduction section.

**Response:** We acknowledge your observation regarding the detailed literature review. We have now condensed this section and transferred only the most salient and relevant background information to the Introduction section, ensuring a more concise and focused literature review.

Hypothesis and variable definitions should be shown as a separate section rather than part of the literature review.

**Response:** In accordance with your recommendation, we have moved the hypotheses and variable definitions into a separate section following the literature review for better clarity and organization.

Authors need to follow the guidelines of PLOS ONE.

**Response:** The manuscript has been thoroughly revised to ensure full compliance with PLOS ONE submission requirements.

For 3.2 Sample: Why is the sampling method Simple Random Sampling? There is no sampling frame; it cannot be a random sample, but it may be convenience sampling because data is collected from respondents who reply via a Google form.

**Response:** We appreciate your correction regarding the sampling method. Since no complete sampling frame was available and data were collected through responses to a Google Form, we have amended the methodology to correctly state that convenience sampling was employed.

In Table 2, what does the term “experience” mean? This table should be shown in the Results section.

**Response:** We have revised the term “experience” in Table 2 to clearly specify that it refers to working experience in the healthcare sector. Additionally, as suggested, Table 2 has been moved from the Methodology section to the Results section.

Discussion points for H5 are necessary to add.

**Response:** We appreciate your attention to the discussion of Hypothesis 5. We would like to clarify that the discussion of H5 is already included in the manuscript (see Discussion section, 3^rd^ and 4^th^ para). However, we have reviewed this section again to ensure it is sufficiently clear and directly linked to the results, adding minor clarifications where needed for better reader comprehension.

**In case of** 3. Have the authors made all data underlying the findings in their manuscript fully available?

The PLOS Data policy requires authors to make all data underlying the findings described in their manuscript fully available without restriction, with rare exception (please refer to the Data Availability Statement in the manuscript PDF file). The data should be provided as part of the manuscript or its supporting information, or deposited to a public repository. For example, in addition to summary statistics, the data points behind means, medians and variance measures should be available. If there are restrictions on publicly sharing data—e.g. participant privacy or use of data from a third party—those must be specified.

Reviewer #1: No

Reviewer #2: Yes

**Response to Reviewer #1:** We thank the reviewer for raising this important point regarding data availability. We confirm that all data underlying the findings of this study are now fully available in accordance with the PLOS ONE Data Policy. The Data Availability Statement in the revised manuscript has been updated accordingly. No restrictions apply to the use of these data.

Reviewer #2: Great work authors.
Sorry for the delay in giving the feedback.
AMR needs to be controlled as soon as possible.
Methodology is clear.
Results are based on the data.
Figures are clear.
Strengths and limitations have been discussed.

**Response to Reviewer #2:**

We sincerely thank the reviewer for the positive and encouraging feedback on our work. We appreciate your acknowledgment of the clarity of our methodology, the data-driven nature of our results, and the quality of our figures. We are also grateful for your recognition that the strengths and limitations have been appropriately discussed. We agree with your remark on the urgent need to control AMR and hope that our study will contribute meaningfully to addressing this pressing global health challenge.
